# Associations between serum adipocytokines and glycemic tolerance biomarkers in a rural Chinese population

**DOI:** 10.1371/journal.pone.0182273

**Published:** 2017-08-07

**Authors:** Xiaoxia Li, Yi Zhao, Yanan Jin, Tianjing Zhang, Xiaoyu Chang, Sha Liao, Hongxia Xu, Xiuying Liu, Jianjun Yang, Jianjun Zhang, Yuhong Zhang

**Affiliations:** 1 Department of Epidemiology, School of Public Health and Management, Ningxia Medical University, Yinchuan, Ningxia Hui Autonomous Region, People’s Republic of China; 2 Ningxia Center for Disease Control and Prevention, Yinchuan, Ningxia Hui Autonomous Region, People’s Republic of China; 3 Sichuan Provincial Center for Disease Control and Prevention, Chengdu, Sichuan Province, People’s Republic of China; 4 Department of Epidemiology, Richard M. Fairbanks School of Public Health, Indiana University, Indianapolis, Indiana, United States of America; East Tennessee State University, UNITED STATES

## Abstract

Although experimental studies have shown that adiponectin and leptin modulate glucose tolerance and insulin resistance, it remains unclear whether these adipocytokines exert similar effects in general human populations. We evaluated the associations of serum adiponectin and leptin with β-cell function and insulin resistance in a population with low obesity prevalence. A cross-sectional study of 783 rural residents, aged 25–74 years, recruited in Ningxia, China was conducted during 2008–2012. β-cell function and insulin resistance were estimated using the Homeostasis Model Assessment. Serum adiponectin and leptin were measured with ELISA. Serum adiponectin concentrations (mean ± SD) were highest in subjects with normal glucose tolerance (36.65 ± 61.13 μg/ml), intermediate in those with impaired fasting glucose (25.92 ± 34.48 μg/ml), and lowest in those with diabetes (15.08 ± 12.14 μg/ml) (p = 0.001). A similar pattern of differences was found for β-cell function, whereas opposite results were observed for insulin resistance and blood glucose. After adjustment for confounders including metabolic syndrome components, serum adiponectin (μg/ml) was inversely associated with β-cell function (%β) [β (95% CI): -7.57 (-12.33, -2.81)] and insulin resistance (100/%S) [β (95% CI): -0.21 (-0.33, -0.09)]. A significant inverse association also existed between serum leptin and β-cell function, but serum leptin was not significantly associated with insulin resistance. The present study suggests that adiponectin and leptin play a role in the development of insulin resistance and diabetes independent of metabolic syndrome.

## Introduction

The global prevalence of overweight, obesity, and diabetes has reached epidemic proportions and thus poses a tremendous public health problem [1, 2]. The World Health Organization has estimated that 39% of the world adult population were overweight and 13% of them were obese in 2014 [3]. The combined prevalence rates of overweight and obesity have risen by 25.5% for adults and 47.1% for children from 1980 to 2013, with a corresponding increase in the number of overweight and obese individuals from 857 million to 2.1 billion during the same period of time [1]. Although obesity prevalence has levelled off in developed countries since 2006, it has been continuously increasing in developing countries, including China [1, 4]. An overall upward trend in the prevalence of diabetes has occurred between 1980 and 2014 primarily due to a simultaneous increase in the prevalence of overweight and obesity [2]. Population-based studies have reported that there were 422 million cases of diabetes in 2014 in the world [2] and 114 million projected cases of diabetes in 2010 in China [5].

A substantial body of epidemiologic and experimental evidence has revealed that overweight and obesity are associated with an elevated risk of insulin resistance and diabetes [6, 7]. Insulin resistance is an adverse health condition in which muscle, fat, and liver cells have a reduced sensitivity to insulin. Numerous studies have found that insulin resistance plays a critical role in the mechanistic pathway linking obesity to diabetes and accounts for the major components of metabolic syndrome (i.e. high serum glucose, high serum triglycerides, and low high-density lipoprotein cholesterol) [7, 8]. The association between obesity and diabetes and its biological mechanisms have gained further support from the intervention studies in which weight reduction improved insulin sensitivity [9] and reduced diabetes risk [10].

Adipocytokines (e.g. adiponectin and leptin) are excreted by adipose tissue and are involved in the homeostasis of energy and regulation of glucose and lipid metabolism [11]. Adiponectin is a hormone that sensitizes the human body to insulin. It has been found that circulating concentrations of adiponectin were decreased in the subjects who were obese or resistant to insulin [11, 12] and obese rhesus monkeys that were at high risk for diabetes [13]. Moreover, elevated adiponectin levels were associated with a reduced risk of diabetes in many prospective cohort studies [14]. Leptin suppresses appetite and enhances energy expenditure. Circulating levels of leptin were elevated in the individuals who were obese and had high insulin levels [15]. Animal and intervention studies suggest that aforementioned alterations in adiponectin and leptin precede the occurrence of insulin resistance and subsequent diabetes [11, 14, 16]. While adiponectin-deficient mice developed hepatic insulin resistance [16], administrating adiponectin or upregulating its plasma concentrations substantially improved insulin sensitivity in the liver and skeletal muscle of animals or humans [11, 14]. Similar results have been obtained for leptin [17–19]. To better elucidate the role of adipocytokines in the development of insulin resistance and diabetes, it is thus critical to investigate the associations of adiponectin and leptin with insulin resistance because most previous studies have focused on evaluating the associations of these adipocytokines with obesity and diabetes [14, 20, 21]. Therefore, the present study sought to examine the associations of serum adiponectin and leptin with β-cell function and insulin resistance among a large sample of the rural Chinese population whose prevalence rates of overweight and obesity are substantially lower than those of Western populations largely due to differences in dietary and lifestyle factors [22].

## Subjects and methods

### Study population

A cross-sectional study was carried out from 2008 to 2012 in three counties or administrative equivalents (i.e. Yuanzhou district, Qingtongxia City, and Pingluo County) in Ningxia Hui Autonomous Region of China. A total of six villages were recruited using stratified cluster sampling according to economic development levels, with two villages (one for Han ethnicity and one for Hui ethnicity) selected from each of the three counties considered. During the study period, 4614 subjects, aged 24–75 years, were enrolled to the study. Pregnant or breastfeeding women and subjects with severe chronic diseases were excluded from the study. Also excluded were subjects who had acute respiratory infection or chronic viral infection and those who underwent surgery in the preceding week of the study recruitment. Of the 4614 subjects who were eligible for the present study, 2393 donated a blood sample to the study. Of the 2393 subjects, 1000 were selected by using mechanical sampling (k = 1) for measuring selected biochemical indicators due to budget constraints. Of the 1000 subjects, serum adipocytokines were determined among 800 subjects due to the depletion of serum samples for the remaining 200 subjects. Of the 800 subjects, six had serum levels of glucose or insulin that were not within the range of clinically plausible values used for calculating the Homeostasis Model Assessment (HOMA) parameters, and 11 subjects were lack of data on insulin concentrations that were not measured due to insufficient amounts of serum samples. After excluding those 17 subjects, valid and complete data from 783 subjects were available for our data analysis. The present study was approved by the Medical Ethics Review Committee of Ningxia Medical University and written informed consent was obtained from all study subjects.

### Subject interview

Subjects were interviewed by trained research assistants with a validated questionnaire that solicits information on age, sex, ethnicity, education, cigarette smoking, alcohol intake, history of disease, etc. The questionnaire was administered by the research assistants, and the completed questionnaire was then checked for its accuracy and validity at the end of the interview.

### Anthropometric measurements

Body height and waist circumference were measured with a portable ruler (accurate to within 0.1 cm) and body weight was quantified with a weight scale (accurate to within 0.1 kg). Body mass index (BMI) was calculated as weight (kg)/height (m^2^). Blood pressure was measured for three times after subjects had rested for at least 30 minutes, and the three readings were averaged and used in data analysis.

### Biochemical measurements

Blood samples were drawn from the subjects at 6:00–8:00 am on the date of interview after 8–12 hours of fasting and alcohol avoidance. Serum samples were separated from the whole blood on the field, transported in an ice-packed box to the local Centers for Disease Control and Prevention within two hours of blood collection (temporarily kept in a freezer at -20°C), then delivered to Ningxia Medical University, and kept in a freezer (-80°C) until time of analysis. Blood glucose was determined by One Touch Ultra 2 (Life Scan, Wayne, PA, USA) immediately after blood was drawn. Serum insulin was measured by enzyme-linked immune chemiluminescence method. Serum high-density lipoprotein (HDL) cholesterol, total cholesterol, and triglycerides were quantified using an automatic biochemical analyzer (COBAS 501, Roche Diagnostics, Switzerland). Serum low-density lipoprotein (LDL) cholesterol was calculated with the Friedewald formula [23]. Serum adiponectin and leptin were measured using commercially available enzyme-linked immunosorbent assay (ELISA) kits (CUSABIO, China) according to the manufacturer’s instructions, with the limit of detection being 100 ng/ml for adiponectin and 400 pg/ml for leptin. For the measurements of both serum adiponectin and leptin, the intra- and inter-assay coefficients of variation (CV) were 8% and 10%, respectively. The intra- and inter-assay CVs were 10% or less for other measured biochemical assays.

### Statistical analysis

β-cell function (HOMA-ß, %ß) and insulin resistance (HOMA-IR, 100/%S) were calculated from fasting blood glucose and serum insulin with the HOMA-2 calculator (http://www.dtu.ox.ac.uk/homacalculator/). As HOMA-IR is simply the reciprocal of HOMA-S, only the former was analyzed in the present study. All subjects were divided into three groups defined by the guidelines of the American Diabetic Association: normal glucose tolerance (fasting blood glucose: <5.6 mmol/L), impaired fasting glucose (fasting blood glucose: 5.6–6.9 mmol/L), and diabetes (fasting blood glucose: ≥7.0 mmol/L, previously diagnosed as diabetes, or currently taking diabetic medications). Differences in demographics, lifestyle, anthropometrics, blood lipids, serum adipocytokines, and HOMA parameters were compared among the three groups using analysis of variance for continuous variables and Chi-square test for categorical variables. The purpose of these comparisons is to investigate whether there are statistically significant differences in these factors among subjects with various degrees of glucose tolerance.

The associations of serum adiponectin and leptin with β-cell function and insulin resistance were evaluated by both multivariable linear regression and logistic regression. For each of the associations considered, two models were constructed: the model 1 adjusted for age, sex, ethnicity, education, cigarette smoking, and alcohol consumption, and the model 2 adjusted for all the variables included in the model 1 as well as the five individual components of metabolic syndrome [i.e. waist circumference, systolic blood pressure, triglycerides, HDL cholesterol, and blood glucose]. While the variables controlled for as confounders in the model 1 were primarily selected on the basis of the prior knowledge of their potential influence on the associations of interest, additional adjustment for metabolic syndrome components in the model 2 was to evaluate whether and to what extent the associations between serum adiponectins examined and the HOMA parameters calculated were mediated through metabolic syndrome.

In the logistic regression analysis, odds ratio (OR) and 95% confidence interval (CI) for the higher levels (>median) of β-cell function or insulin resistance were calculated for subjects who were in the second, third, and fourth quartile of each of these two parameters, compared with those who were in the respective first (lowest) quartile. Linear trend across quartiles of adiponectin and leptin was evaluated using the median in each quartile to create an ordinal variable and entering it into regression models. As the study population is composed of subjects from two ethnicities (Han and Hui), potential interactions of ethnicity with adiponectin and leptin on β-cell function and insulin resistance were tested. As none of the interaction terms tested was statistically significant, ethnicity-specific analysis was not performed. The SPSS (version 14.0, SPSS Corp, College Station, TX) was used for statistical analyses. A p-value of <0.05 was considered statistically significant.

## Results

The characteristics of study subjects were compared among the groups of normal glucose tolerance (n = 376), impaired fasting glucose (n = 362), and diabetes (n = 45) in Table 1. Individuals with diabetes were older and less likely to be male than those with normal glucose tolerance or impaired fasting glucose. A gradient increase was observed in BMI, waist circumference, blood pressure, serum total cholesterol, and serum triglycerides across the three groups compared (all p <0.01).

**Table 1:** Characteristics of study subjects by different levels of glycemic tolerance in a rural Chinese population, 2008–2012.

Characteristics	All Subjects (n = 783)	Subjects with NGT (n = 376)	Subjects with IFG (n = 362)	Subjects with DM (n = 45)	P value
Age (years)	49.3±11.5	48.5±11.8^a^	49.5±11.2	54.5±11.5^c^	0.004
Sex, male (%)	44.2	50.0^a^	40.9^b^	22.2^c^	<0.0001
Ethnic group (%)					
Han	55.7	53.5	58.3	53.3	0.40
Hui	44.3	46.5	41.7	46.7	
Education (%)					
Illiteracy	42.0	41.2	40.1	64.4	
Elementary School	28.0	29.5	27.3	20.0	0.03
Other	30.0	29.3	32.6	15.6	
Cigarette Smoking (%)	18.4	21.3	16.0	13.3	0.33
Alcohol Drinking (%)	11.7	11.2	12.7	8.9	0.54
Body Mass Index (kg/m^2^)	23.6±3.3	23.0±3.2^a^	23.9±3.3^b^	25.6±3.7^c^	<0.0001
Waist Circumference (cm)	81.5±9.8	79.6±9.6^a^	82.6±9.4^b^	89.8±9.4^c^	<0.0001
Systolic blood pressure (mmHg)	124±19	122±19^a^	125±19^b^	144±18^c^	<0.0001
Diastolic blood pressure (mmHg)	79±11	77±11^a^	79±12^b^	87±11^c^	<0.0001
Total cholesterol (mmol/L)	4.08±0.90	3.98±0.86^a^	4.11±0.88^b^	4.63±1.07^c^	<0.0001
Triglycerides (mmol/L)	1.58±1.18	1.37±0.76^a^	1.63±1.00^b^	2.92±3.02^c^	<0.0001
HDL-Cholesterol (mmol/L)	1.29±0.33	1.30±0.33	1.28±0.32	1.21±0.35	0.25
LDL-Cholesterol (mmol/L)	2.08±0.71	2.06±0.71	2.09±0.69	2.15±0.83	0.68

NGT, normal glucose tolerance; IFG, impaired fasting glucose; DM, diabetes mellitus; HDL, high-density lipoprotein; and LDL, low-density lipoprotein

Values shown are mean ± standard deviation for continuous variables

^a, b and c^ indicates p<0.05 for group comparisons: a for NGT vs. DM, b for NGT vs. IFG and c for IFG vs. DM.

Differences in serum adipocytokines, blood glucose, serum insulin, β-cell function, and insulin resistance among the three groups were shown in Table 2. Serum adiponectin concentrations (mean ± SD) were highest in subjects with normal glucose tolerance (36.65 ± 61.13 μg/ml), intermediate in those with impaired fasting glucose (25.92 ± 34.48 μg/ml), and lowest in those with diabetes (15.08 ± 12.14 μg/ml) (p = 0.001). A similar pattern of differences was found for β-cell function, whereas an opposite pattern of differences was observed for insulin resistance and blood glucose. No significant differences in serum leptin existed among the three groups considered.

**Table 2:** Differences in serum concentrations of adipocytokines, blood glucose, insulin, and HOMA parameters among 783 rural Chinese residents with different levels of glycemic tolerance in Ningxia, 2008–2012.

Variables	Subjects with NGT (n = 376)	Subjects with IFG (n = 362)	Subjects with DM (n = 45)	P value
Serum Adiponectin (μg/ml)	36.65±61.13^a^	25.92±34.48^b^	15.08±12.14	0.001
Serum Leptin (ng/ml)	8.42±8.65	8.72±8.48	11.30±15.41	0.13
Fasting Blood Glucose (mmol/L)	5.2±0.3^a^	6.0±0.4^b^	9.2±3.7^c^	<0.0001
Fasting Serum Insulin (pmol/L)	38.76±27.29^a^	47.94±63.91^b^	71.23±71.92^c^	<0.0001
β-Cell Function (%β)	70.0±29.9^a^	59.0±39.0^b^	44.0±37.0^c^	<0.0001
Insulin Resistance (100/%S)	0.73±0.5^a^	0.92±1.0^b^	1.54±1.4^c^	<0.0001

NGT, normal glucose tolerance; IFG, impaired fasting glucose; and DM, diabetes mellitus

Values shown are mean ± standard deviation

^a, b and c^ indicates p<0.05 for group comparisons: a for NGT vs. DM, b for NGT vs. IFG and c for IFG vs. DM.

Table 3 showed that serum levels of adiponectin (μg/ml) were significantly inversely associated with β-cell function (%β) [β (95% CI): -7.57 (-12.33, -2.81)] and insulin resistance (100/%S) [β (95% CI): -0.21 (-0.33, -0.09)] after adjustment for confounders. A significant inverse association was also observed between serum leptin and β-cell function, but serum leptin concentrations were not significantly associated with insulin resistance. However, the statistically significant inverse associations of serum adiponectin and leptin with β-cell function disappeared after additional adjustment for insulin resistance (data not shown).

**Table 3:** Multiple linear regression analysis of the associations between serum adipocytokines and HOMA parameters among 783 rural Chinese residents in Ningxia, 2008–2012.

Independent variables	β-Cell function (%ß)		Insulin Resistance (100/%S)	
	β	95% CI	β	95% CI
Serum adiponectin (μg/ml)*				
Model 1	-6.01	(-10.51, -1.52)	-0.29	(-0.40, -0.18)
Model 2	-7.57	(-12.33, -2.81)	-0.21	(-0.33, -0.09)
Serum Leptin (ng/ml)*				
Model 1	-5.87	(-10.70, -1.03)	-0.05	(-0.17, 0.06)
Model 2	-5.16	(-9.85, -0.47)	-0.07	(-0.18, 0.05)

Abbreviations: β, partial regression analysis; CI: confidence interval

* Log-transformed values were used in the analysis

Model 1: Adjusted for age, sex, ethnic group, education, cigarette smoking, and alcohol consumption Model 2: adjusted for all those confounders in the model 1 as well as waist circumference, systolic blood pressure, triglycerides, high density lipoprotein cholesterol, and blood glucose.

The results of the logistic regression analysis for the risk of having the higher level (≥median) of each of the two HOMA parameters examined in relation to adipocytokines were presented in Table 4. The findings obtained from the logistic regression analysis were overall similar to those derived from the multiple linear regression analysis. OR (95% CI) for the higher level of β-cell function was 0.46 (0.26, 0.79) (p-trend <0.0001) for comparing the fourth quartile with first quartile of serum adiponectin. The corresponding OR (95% CI) was 0.55 (0.33, 0.92) (p-trend <0.037) for serum leptin. Elevated concentrations of serum adiponectin were associated with a reduced risk of developing the higher level of insulin resistance, but this association was no longer significant after adjustment for the five components of metabolic syndrome.

**Table 4:** Odds ratios (OR) and 95% confidence intervals (CI) for the higher levels (≥ median) of the HOMA parameters in relation to serum concentrations of adipocytokines in a rural Chinese population in Ningxia, 2008–2012.

Adipocytokines	No. of Subjects < and ≥ median	β-Cell function (%ß)		Insulin Resistance (100/%S)	
		Model 1	Model 2	Model 1	Model 2
Adiponectin (μg/ml)					
Q1 (<10.7)	97/96	1	1	1	1
Q2 (10.7-< 18.4)	100/96	1.00 (0.64, 1.54)	0.98 (0.59, 1.62)	0.95 (0.62, 1.46)	0.93 (0.59, 1.48)
Q3 (18.6- <35.6)	99/99	1.30 (0.84, 2.02)	1.29 (0.77, 2.17)	0.61 (0.40, 0.93)	0.80 (0.50, 1.26)
Q4 (≥35.6)	98/98	0.62 (0.40, 0.95)	0.46 (0.26, 0.79)	0.37 (0.24, 0.56)	0.64 (0.39, 1.06)
*P* for trend		0.011	<0.0001	<0.0001	0.08
Leptin (ng/ml)					
Q1 (<4.1)	95/96	1	1	1	1
Q2 (4.1- < 6.6)	98/102	0.72 (0.47, 1.12)	0.74 (0.45, 1.22)	0.77 (0.51, 1.16)	0.74 (0.48, 1.15)
Q3 (6.6- <11.1)	100/100	0.47 (0.30, 0.72)	0.52 (0.32, 0.84)	0.68 (0.45, 1.04)	0.61 (0.39, 0.95)
Q4 (≥11.1)	96/96	0.49 (0.31, 0.77)	0.55 (0.33, 0.92)	0.78 (0.50, 1.21)	0.71 (0.44, 1.13)
*P* for trend		0.002	0.037	0.44	0.19

Model 1: Adjusted for age, sex, ethnic group, education, cigarette smoking, and alcohol consumption; Model 2 adjusted for all those confounders in the model 1 as well as waist circumference, systolic blood pressure, triglycerides, high density lipoprotein cholesterol, and blood glucose.

## Discussion

The major findings of the present study were that there was a significant gradient decrease in serum concentrations of adiponectin across the three groups of subjects with different levels of glycemic tolerance (i.e. normal glucose tolerance, impaired fasting glucose, and diabetes). Both serum adiponectin and leptin were inversely associated with β-cell function, whereas only serum adiponectin was inversely associated with insulin resistance. Of note, the inverse associations of serum adipocytokines examined with β-cell function were no longer significant after insulin resistance was further adjusted in the multiple linear regression analysis.

Our observation that circulating concentrations of adiponectin were lower in the subjects who were pre-diabetic (i.e. impaired fasting glucose) or diabetic than in the subjects who maintained normal glucose tolerance was generally consistent with the results of previous studies [14, 24–28]. A significant reduction in serum adiponectin levels among obese, pre-diabetic, and diabetic individuals in comparison with healthy subjects have been found in several studies carried out in populations with different dietary and lifestyle habits [25–27, 29]. In a British study of 150 South-Asians, serum adiponectin concentrations were highest in subjects with normal glucose tolerance, reduced in those with impaired glucose tolerance, and were lowest in those with diabetes [29], which essentially confirmed the results obtained from 783 Chinese residents in the present study. However, this pattern of difference in serum adiponectin levels was not observed in some other studies, including a study among 92 Yemeni volunteers [30].

A meta-analysis of 13 prospective cohort studies published in 2009 showed an inverse monotonic association between adiponectin levels and the risk of type 2 diabetes [relative risk (95% CI): 0.72 (0.67–0.78) per 1–log μg/mL increment in adiponectin levels], although most of those studies were conducted in Western populations with a high prevalence of obesity [14]. Subsequent epidemiologic studies have yielded similar results [25]. The present study is different from previous studies in that our observed significant associations of serum adiponectin with β-cell function and insulin resistance were independent of not only demographic, socioeconomic, and lifestyle factors but also the individual components of metabolic syndrome.

Animal and experimental studies have offered several lines of biological plausibility for the associations of circulating adiponectin with insulin resistance and diabetes. It appears that decreased adiponectin concentrations increase the risk of developing insulin resistance and ensuing diabetes. Adiponectin knockout mice developed hepatic but not peripheral insulin resistance, which was evaluated with the euglycemic insulin clamp technique [16]. Adiponectin administration ameliorated insulin resistance in lipoatrophic mice and type 2 diabetic mice [31] and reduced blood glucose levels in normal mice [32]. In addition, it has been proposed that the insulin-sensitizing and antidiabetic actions of thiazolidinediones are at least in part mediated through their induced increase in circulating adiponectin levels [11]. Binding of adiponectin to its receptors (adipoR1 and adipooR2) activates p38 MAPK, AMPK, and PPARα signaling pathways, leading to increased glucose uptake in muscle, decreased gluconeogenesis in the liver, and increased fatty acid oxidation in the liver and muscle, respectively [11]. The present study showed that the inverse association between serum adiponectin concentrations and insulin resistance became somewhat weaker after adjustment for metabolic syndrome components, which suggests that these associations are partially but not entirely confounded by this abnormal health condition.

Mounting evidence suggests that leptin is implicated in glycemic tolerance independent of its effect on energy balance [17, 33]. It was reported that systematic infusion of leptin reversed insulin resistance in congenital lipodystrophic mice characterized by hyperinsulinemia and hyperglycemia [19]. Similar results were obtained from patients with severe lipodystrophy in which chronic leptin treatment improved hepatic and peripheral insulin resistance [18]. In the present study, we found that higher serum concentrations of both adiponectin and leptin were inversely associated with β-cell function. As the β-cell function calculated from the HOMA model reflects β-cell activity (i.e. insulin secretion) rather than its health or pathological condition [34], these inverse associations indicate reduced demand for insulin secretion in response to elevated plasma levels of adiponectin and leptin. The disappearance of the significant associations of serum adiponectin and leptin with β-cell function after additional adjustment for insulin resistance suggests that this reduced demand for insulin section is largely driven by enhanced insulin sensitivity arising from increased levels of the adipocytokines examined.

The associations of leptin levels with insulin resistance and diabetes are overall inconsistent as the positive, inverse, and null associations have been reported previously [15, 26, 33]. Our study revealed no statistically significant association between serum leptin and insulin resistance in both multiple linear and logistic regression analyses, a finding also observed in a nested case-control study in an Indian population [25]. A meta-analysis of 11 prospective cohort studies showed that diabetes risk significantly increased by 37% per 1-log ng/mL increment in leptin levels in men but not in women [35]. However, it should be noted that the subjects recruited to 10 of the 11 studies analyzed were of European origin. The potential reasons for these discrepant results on the effect of leptin concentrations on prediabetes and diabetes across epidemiologic studies may be ascribed to their differences in obesity and diabetes prevalence of study populations, the validity of study design and data collection, and the adequacy of confounding control.

There are some advantages in the present study. We investigated the associations of circulating adipocytokines with β-cell function and insulin resistance among 783 normal, prediabetic, and diabetic subjects, a sample size that is larger than that of most previous cross-sectional studies [25, 30, 33]. Our study was conducted among a rural Chinese population that has lower prevalence rates of obesity and diabetes than those of Western populations where the majority of published studies on the same topic have been conducted. Therefore, the present study offers timely and highly needed data on the associations between serum adipocytokines and glycemic tolerance in a population with a relatively low risk of prediabtics and diabetes. Furthermore, the associations reported in this paper were controlled for suspected and established confounders.

The present study is subject to several weaknesses. Blood glucose levels and serum concentrations of insulin, adiponectin, and leptin were measured only once. Therefore, it is possible that some subjects might have been misclassified with regard to the usual levels of these biomarkers due to their potential daily variations, which could lead to attenuated estimates of the true associations. As insulin secretion is pulsatile, insulin concentrations measured with a single blood sample may vary with the time of sample collection. However, a study has showed acceptable intra-subject coefficients of variation for HOMA parameters (7.7% for β-cell function and 10.3% for insulin sensitivity) [36]. In our study, β-cell function and insulin resistance were not measured using the euglycemic clamp or glucose tolerance test, which is the gold standard for these assays [37] but is not feasible for application in large epidemiologic studies. Nevertheless, modest to strong correlations have been reported between estimates of β-cell function (R_s_ or r = 0.62–0.90) and insulin sensitivity (R_s_ or r = 0.58–0.88) derived from the HOMA model and from the euglycemic clamp [34]. Of the 45 diabetic patients included in the present study, 11 received exogenous insulin, which could have somewhat distorted their values of β-cell function calculated from the HOMA model. However, adjustment for use of exogenous insulin did not materially alter the main results obtained.

The data analyzed were collected from a cross-sectional study, which makes it possible for us to draw any causal inference on the associations of serum adipocytokines with β-cell function and insulin resistance. Women generally have higher circulating concentrations of adiponectin than men [11]. This sex difference was considered in our data analysis because sex was adjusted as a confounder in multivariable regression models. As in any epidemiological studies, our study results might have been affected by residual confounding due to unknown, unmeasured, and/or inadequately measured confounders. In addition, the possibility that some of observed positive results might have been observed by chance due to multiple comparisons could not be entirely ruled out.

In summary, the present study revealed that serum concentrations of adiponectin significantly decreased across the spectrum of glycemic tolerance from normal glucose tolerance to impaired glucose tolerance to diabetics in a rural Chinese population. Both serum levels of adiponectin and leptin were inversely associated with β-cell function, but the inverse association with insulin resistance was found only for serum adiponectin. These effects of adipocytokines overall persisted after adjustment for the components of metabolic syndrome. It should be pointed out that serum adiponectin and leptin were no longer significantly associated with β-cell function when insulin resistance was additionally controlled for in the multiple linear regression analysis. The findings of the present study are expected to lead to an improved understanding of the role of adiponectin and leptin in the etiology, pathogenesis, and prevention of prediabetics and diabetics if they can be replicated in other populations with low prevalence of overweight and obesity.

## Supporting information

S1 File This is Excel database about general information, anthropometric measurements and biochemical measurements. (XLS)
